# Modulation of Network Excitability by Persistent Activity: How Working Memory Affects the Response to Incoming Stimuli

**DOI:** 10.1371/journal.pcbi.1004059

**Published:** 2015-02-19

**Authors:** Elisa M. Tartaglia, Nicolas Brunel, Gianluigi Mongillo

**Affiliations:** 1 Center for Neuroscience and Cognitive Systems @UniTn, Istituto Italiano di Tecnologia, Rovereto, Italy; 2 Centre National de la Recherche Scientifique, Paris, France; 3 Université Paris Descartes, Centre de Neurophysique, Physiologie et Pathologie, Paris, France; 4 Departments of Statistics and Neurobiology, University of Chicago, Chicago, Illinois, United States of America; Indiana, United States

## Abstract

Persistent activity and match effects are widely regarded as neuronal correlates of short-term storage and manipulation of information, with the first serving active maintenance and the latter supporting the comparison between memory contents and incoming sensory information. The mechanistic and functional relationship between these two basic neurophysiological signatures of working memory remains elusive. We propose that match signals are generated as a result of transient changes in local network excitability brought about by persistent activity. Neurons more active will be more excitable, and thus more responsive to external inputs. Accordingly, network responses are jointly determined by the incoming stimulus and the ongoing pattern of persistent activity. Using a spiking model network, we show that this mechanism is able to reproduce most of the experimental phenomenology of match effects as exposed by single-cell recordings during delayed-response tasks. The model provides a unified, parsimonious mechanistic account of the main neuronal correlates of working memory, makes several experimentally testable predictions, and demonstrates a new functional role for persistent activity.

## Author Summary

Over short time periods, memories are stored by sustained patterns of spiking activity which, once initiated by the stimulus, persist over the entire retention interval. How the information stored by such persistent activity is later retrieved is presently unclear. Here we propose that, besides temporarily storing memories, persistent activity is also instrumental in their retrieval by transiently modifying the tuning properties of the underlying neuronal networks. We show that the mechanism proposed parsimoniously recapitulates the extensive experimental phenomenology on match effects observed in delayed-response tasks, where the information held in memory has to be compared with incoming, sensory-related information to act appropriately. The theory makes very specific, straightforwardly testable predictions.

## Introduction

Memory allows an organism to store information about past events and then use it to modify its behavior in response to future ones. As a simple example, consider the classic delayed match-to-sample (DMS) task, where the appropriate behavior upon presentation of the test stimulus is conditional on the sample stimulus (the past event). What are the mechanisms that support the *storage* of information about the sample stimulus and its *retrieval* upon the presentation of the test stimulus? Electrophysiological studies have exposed two neuronal correlates of those mechanisms: (i) sample-selective persistent activity during the delay period (see, e.g., [1–3]); (ii) differential test-period activity depending on whether the test stimulus matches or not the sample (see, e.g., [1, 4–7]). Specifically, some neurons show enhanced responses in the match as compared to the non-match condition (match enhancement), while others show the opposite pattern (match suppression), despite the fact that stimuli are physically identical in the two conditions.

Persistent delay activity, together with match effects, are basic neuronal hallmarks of temporary maintenance and manipulation of information. Persistent activity has been primarily associated with short-term storage, while match effects are thought to reflect the outcome of the comparison between stored and incoming information. Consistently with these putative roles, they have been observed across different short-term memory protocols besides the DMS task, such as DMS with intervening distractors [8, 9], delayed non-match-to-sample [10], delayed paired-associate [11], delayed categorization [12] and delayed cue-instructed go/nogo tasks [13], and with different class of stimuli, such as natural images, fractal images, spatial locations, motion directions and pure tones. Persistent activity and match effects have also been observed across diverse cortical regions and, interestingly, the basic phenomenology exposed in regions traditionally associated to memory function, such as the pre-frontal, infero-temporal and parietal cortices [8, 9, 14], is qualitatively very similar to the phenomenology observed in regions traditionally associated to sensory processing, such as auditory cortex [15], area V4 [16] and area MT [17].

It is presently unclear how the information stored in the pattern of persistent activity is retrieved and compared with incoming stimuli. One theory holds that match enhancement and persistent activity on one hand, and match suppression on the other hand, are neuronal manifestations of two distinct, *parallel mechanisms* supporting memory storage and retrieval [1, 18]. Enhancement reflects the operation of an active detector which signals the appearance of behaviorally relevant stimuli, stored by persistent activity during the delay period. Suppression, instead, reflects the operation of an automatic detector which signals stimulus repetitions regardless of their behavioral relevance, and its functioning is assumed to be independent of persistent activity. Current mechanistic modeling fully embraces this notion of *parallel mechanisms* (see, e.g., [19]). Match suppression, which is generally the most prominent effect, is understood as the result of activity-dependent *fatigue*, either at the single-cell or at the synaptic level, produced by the sample presentation. Match enhancement, on the other hand, is understood as the result of selective inputs from a different area, or from a functionally different neuronal sub-population within the same area, carrying information about the stimulus actively held in memory.

In some cases, however, the reported experimental phenomenology does not appear to be fully consistent with the parallel mechanisms theory. In delayed paired-associate tasks, the response is conditional on whether the test stimulus matches the pair-associate of the sample stimulus. No repetition occurs. According to the theory, only match enhancement (or no effect, depending on the identity of the test stimulus) should be observed. Experiments report both enhancement and suppression [11]. In delayed match-to-category tasks where stimuli also do no repeat within a trial, similarly both enhancement and suppression are observed when the test matches the category of the sample [12]. In a recent study using a delayed cue-instructed go/nogo tasks with distractors, differential responses to *neutral* stimuli depending on the cue stimulus have been reported [13]. The theory would rather predict no response modulation, since neutral stimuli have no behavioral relevance nor they repeat during the trial. Finally and importantly, both match enhancement and selective delay-period activity (together with match suppression) have been observed in passive fixation tasks [14, 20–24], where the theory would predict only match suppression. In fact, in such a task none of the stimuli has behavioral relevance and, thus, there is no need for *active* repetition detection, let alone memory maintenance.

**Figure 1:**
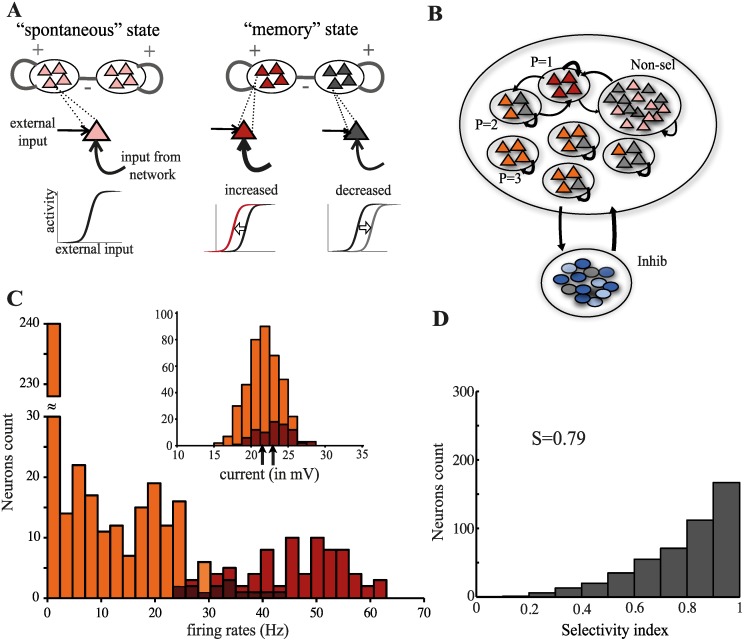
Modulation of tuning properties by network states and evoked-response properties in the spontaneous state. (**A**) The different network states—*spontaneous* (i.e., before stimulus presentation) and *memory* (i.e., following stimulus offset)—are characterized by different distributions of recurrent inputs. In the spontaneous state, neurons in all memory representations are receiving, on average, the same input and are thus firing at the same low, baseline rate (pink). In the memory state, neurons belonging to the memory representation associated with the presented stimulus receive stronger recurrent inputs and thus fire at enhanced rates (red). The activity of neurons belonging to the other memory representations is reduced due to global inhibition (grey). High-firing neurons are more responsive to external inputs, due to increased recurrent inputs, and see their transient response enhanced upon subsequent stimulus presentations (red sigmoidal-like transfer function). Low-firing neurons are less responsive to external inputs, due to reduced recurrent inputs, and see their transient response suppressed upon subsequent stimulus presentations (grey sigmoidal-like transfer function). (**B**) Network architecture. (**C**) Distribution of firing rates upon stimulus presentation in the spontaneous state. Neurons in the memory representation associated with the presented stimulus are shown in red; neurons in the other memory representations are shown in orange. The overlap between the two distributions is labelled in dark red. The inset shows the corresponding distributions of the additional inputs, whose means are indicated by the two arrows. (**D**) Histogram of the sparseness indices in the spontaneous state.

Here, we propose an alternative to the *parallel mechanisms* theory, where match enhancement and suppression both result from a single mechanism: the modulation of network excitability brought about by the persistent activity. Information about the presented stimulus is stored across the delay period by a pattern of persistent activity in which, depending on the stimulus, some neurons increase their spiking rate while others decrease it. Changes in the spiking rate must be accompanied by changes in the neuron’s responsiveness to inputs, whereby, upon presentation of a subsequent stimulus, more active neurons will be more depolarized and thus more excitable, while less active neurons will be more hyperpolarized and thus less excitable [25] (Fig. 1A). Changes in single neuron’s responsiveness entail modifications of the tuning properties of the network, which depend on the ongoing pattern of persistent activity. In our account the differential stimulus-evoked activity depending on previously presented stimuli—i.e., the match effects—is a result of those modifications. We show that the above described mechanism, when implemented in a spiking model network, can reproduce consistently and quite naturally, most of the experimental phenomenology about match effects observed in short-term memory tasks, with no need for *fatigue* mechanisms nor functional differentiation among neurons.

## Results

We model a local cortical circuit involved in memory storage and retrieval using a standard attractor network architecture [26, 27] (Fig. 1B). The network is composed of all-to-all connected excitatory and inhibitory leaky integrate-and-fire neurons. The excitatory sub-network contains *p* = 6 non-overlapping *memory representations*, each corresponding to a different stimulus, as well as neurons that do not participate in the task (non-selective neurons). Each memory representation consists of a fraction *f* = 0.05 of neurons. Synapses between neurons in the same memory representation are stronger than synapses between neurons in different memory representations. Synaptic connectivity in the rest of the network is unstructured. This synaptic organization results in an *effective* inhibitory interaction among the memory representations. Excitatory recurrent inputs have both a fast and a slow component (mimicking NMDA-Rs kinetics), while inhibitory inputs have only a fast component [28]. All neurons also receive non-selective, *noisy* excitatory inputs from outside the local network (background input).

Parameters are chosen so that the network exhibits multi-stability between a *spontaneous activity* state, where all memory representations are active at the same, low firing rate and *p* different *memory* states, where one of the memory representation is active at high rate, while the others are quiescent. The different steady states are attractors of the collective network dynamics. See Methods for details.

### Network response in the spontaneous state

Cortical neurons typically show visual responses to a large faction of stimuli, even after such stimuli have become fairly familiar [29, 30]. To reproduce this feature, we consider that the presentation of a stimulus elicits additional inputs (on top of the background input) to all neurons in the memory representations. Such inputs, for each neuron and for each stimulus, are drawn independently at the beginning of the simulation—and kept fixed thereon—from two Gaussian distributions with same variance but different means. Inputs to neurons in the memory representation associated to the stimulus presented are drawn from the Gaussian with larger mean, while inputs to neurons in the remaining representations are drawn from the Gaussian with smaller mean (inset in Fig. 1C). Means and variance of the two distributions are chosen to qualitatively reproduce the statistics of evoked cortical responses. The resulting firing rates distribution is shown in Fig. 1C. Evoked responses vary in a wide range, from quiescence to relatively high firing rates. The high-rates tail is mostly constituted by neurons in the memory representation of the stimulus being presented (red). However, a significant fraction of neurons in the other memory representations (orange) also fires at high rates. For each neuron in the memory representations, we also measure the *sparseness* of its responses across the stimulus set via the sparseness index (see Methods), which takes on values between 0 (the neuron responds to all stimuli in the same way) and 1 (the neuron responds to a single stimulus). The resulting distribution is shown in Fig. 1D. The average sparseness index as well as its distribution across neurons are consistent with experimental estimates from visual responses evoked in ITC by familiar stimuli [30].

The pattern of stimulus-evoked activity generated by the model reproduces some of the basic features of cortical responses: sparseness at the network level, right-skewed long-tailed distribution of firing rates, and broad tuning curves at the single-cell level [30].

Next, we study how the features of stimulus-evoked activity are modified when the network is in a memory state.

### Network response to repeating stimuli

**Figure 2:**
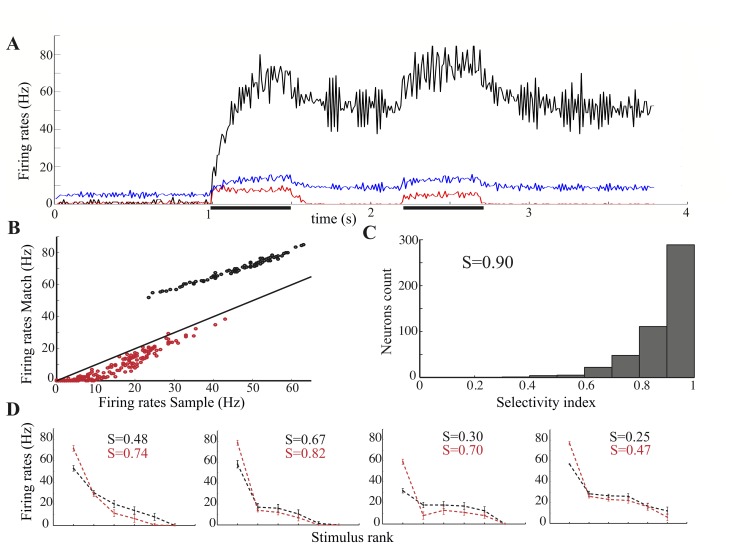
Network response to repeating stimuli (match trial). (**A**) Average activity level in the memory representation associated with the presented stimulus (black), in the other representations (red) and in the inhibitory sub-network (blue). Presentation periods are indicated by the thick horizontal black lines. (**B**) Scatter plot of average single-neuron responses to the first and the second presentation. Responses are calculated by counting the number of spikes during the first 200ms of stimulus presentation. Color code as in (A). (**C**) Histogram of the selectivity indices of the responses to the second presentation upon repeating stimuli. (**D**) Sample single-neuron tuning curves to the first (black) and to the second presentation (red). Stimuli are ranked from best to worst according to the responses to the first presentation (sample).

We consider the case in which the *active* memory representation is the one associated with the incoming stimulus. This corresponds to a match trial in the DMS task. Accordingly, we present the same stimulus twice, with the two presentations separated by a delay period. The first presentation strongly activates the corresponding memory representations (black, Fig. 2A) and, to a lesser extent, all the other representations (red, Fig. 2A) due to the broad selectivity of the evoked responses. At stimulus offset, the memory representation activated by the stimulus remains active at high rates, while the others become quiescent due to the increased level of inhibition (blue, Fig. 2A). Such re-organization of the spiking activity following stimulus presentation is due to a (self-sustained) change in the distribution of the recurrent inputs in the network. Neurons in the active representation receive increased inputs as a result of the positive feedback via the strengthened recurrent synapses. The other neurons receive decreased inputs as a result of the negative feedback due to global inhibition. As a consequence, neurons in the active/inactive representation have a higher/smaller response to stimulus repetition (Fig. 2A). Although the basic features of stimulus-evoked activity in the spontaneous state are qualitatively preserved in the memory state, significant quantitative changes occur. In Fig. 2B we plot the response to the first presentation (sample) *vs.* the second presentation (match) for the neurons in the active (black) and inactive (red) memory representations. All neurons in the active representation show enhanced responses to the second presentation, although to a different extent. Neurons that show the largest relative increase are those that had moderate responses upon sample presentation. Neurons in the inactive representations also show a wide range of suppressed responses to the second presentation. Neurons with weak responses upon sample presentation (i.e. a significant fraction of the responsive neurons—see Fig. 1C) show no response at all or very strong suppression upon match presentation. Neurons with moderate-to-strong responses show modest levels of suppression or no suppression at all. The memory-dependent modulation of single-neuron responsiveness entails a strong increase in the selectivity of the responses evoked by the second presentation (compare the distributions of the sparseness index in Fig. 2C and Fig. 1D). In our model, the best stimulus for a neuron is typically the one for which the neuron exhibits mnemonic activity so that, upon repetition of that stimulus, its response significantly increases. For a different stimulus the neuron does not exhibit mnemonic activity and thus, upon repetition, its response is suppressed. This is clearly seen in Fig. 2D where we show four single-neuron tuning curves. Stimuli are ranked from best to worst according to the evoked response at sample presentation, i.e. when the network is in the spontaneous state (in black). Upon repetition, i.e. when the network is in the corresponding memory state, the response to the best stimulus increases significantly, while those to the other stimuli decrease (in red). Note the amount of sharpening as quantified by the sparseness indices.

### Network response to non-repeating stimuli

**Figure 3:**
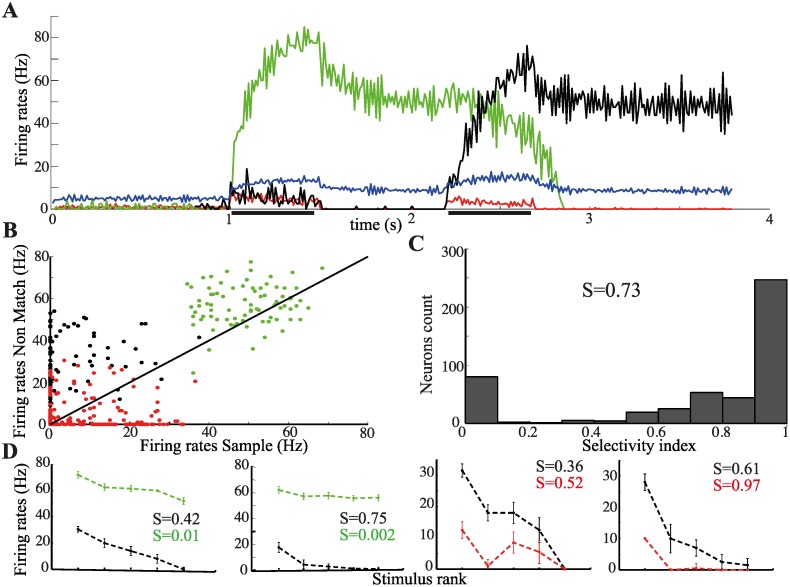
Network response to non-repeating stimuli (non-match trial). (**A**) Average activity level in the memory representation associated with the first stimulus (green), with the second stimulus (black), in the other representations (red) and in the inhibitory sub-network (blue). Presentation periods are indicated by the thick horizontal black lines. (**B**) Scatter plot of average single-neuron responses to the first and the second presentation. Responses are calculated by counting the number of spikes during the first 200ms of stimulus presentation. Color code as in (A). (**C**) Histogram of the selectivity indices of the responses to the second presentation upon non-repeating stimuli. (**D**) Single-neuron tuning curves to the first (black) and to the second presentation for neurons in the active representation (green) and in the non-active representations (red). Stimuli are ranked from best to worst according to the responses to the first presentation (sample).

We consider the case in which the incoming stimulus is associated with a memory representation different from the one currently active. This corresponds to a non-match trial in the DMS task. Accordingly, we present two different stimuli separated by a delay period. This case requires the consideration of three different neuronal sub-populations: neurons belonging to the active representation, neurons belonging to the representation associated to the incoming stimulus, and neurons in the remaining representations. The time course of the average activity in these sub-populations (together with the inhibitory population—blue line) is shown in Fig. 3A. The presentation of the first stimulus (sample) activates the corresponding memory representation (green), which then stays active during the delay period much as before (Fig. 2A). The presentation of the second stimulus (non-match) evokes a strong response in the corresponding memory representation (black), which eventually shuts down the mnemonic activity related to the first stimulus. The response to the second stimulus is, however, smaller than the response to the first stimulus due to the increased level of inhibition when the network is in a memory state. The neurons not belonging to the active representation nor to the one associated with the non-match exhibit suppressed responses to the second presentation (red). This can be also seen in the distribution of red dots in the scatter plot in Fig. 3B. Interestingly, neurons in the representation active during the first delay (green dots) give a strong, transient response to the second stimulus, which is typically larger than the response evoked by the presentation of the associated stimulus (i.e., the sample). During the second presentation, these neurons receive additional inputs which, although smaller than the ones received by neurons in the representation of the stimulus currently presented (black dots), elicit a strong response, due to the increased responsiveness brought about by persistent activity. These additional inputs are statistically the same as the inputs to all the remaining representations, nevertheless, they elicit dramatically different responses (compare green and red dots in Fig. 3B). This most clearly illustrates the importance of the current network state in determining the responses to incoming stimulation.

In Fig. 3C we report the distribution of the sparseness index measured by taking out the stimulus whose memory representation is active during the delay period. The distribution is bimodal as a consequence of the increased responsiveness of neurons in the active representation, which transiently respond to all stimuli, inducing a loss of selectivity. On the other hand, the selectivity of the neurons not belonging to the active representation is sharpened as a result of the suppression of weak/moderate responses. Such differential changes in selectivity are clearly shown in the two sets of tuning curves in Fig. 3D.

### Match-Non Match effects

**Figure 4:**
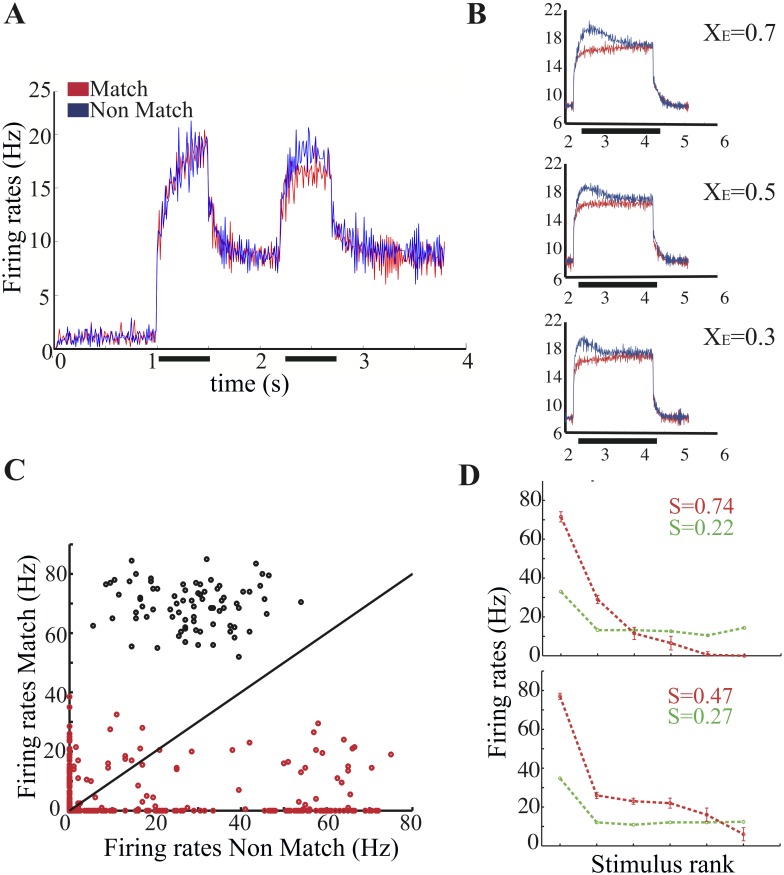
Match vs non-match effects. (**A**) Average activity in the memory representations during match and non-match trials. (**B**) Average activity in the memory representations for a longer match/non-match presentation time (2s) and for three different values of the fraction of slow recurrent inputs *X*_*E*_. Note that the top panel in B and panel A differ only in the presentation time and in the number of trials run -20 trials for each protocol in the former, a single exemplary trial for each protocol in the latter. (**C**) Scatter plot of average single-neuron responses to match and non-match presentation. Responses are calculated by counting the number of spikes during the first 200ms of stimulus presentation. Color code as in Fig. 2B. (**D**) Sample single-neurons tuning curves in match (red) and non-match (green) conditions.

In line with the analysis commonly performed on neurophysiological data, we average neuronal responses, across all neurons and all stimuli, conditional on whether responses are collected in match or non-match trials (e.g. [8, 9, 24]). In Fig. 4A we plot the average activity across memory representations *vs.* time in match and non-match trials. Consistently with recordings in ITC and PFC [8, 11], we find an overall suppression effect: the network response is weaker in match than in non-match condition. However, the scatter plot in Fig. 4C shows that while the largest proportion of cells have a stronger response to non-match than to match (red dots below the diagonal), there are fewer cells (black dots) which show the opposite effect [8, 9, 11, 24, 31]. Interestingly, in our model, cells which show match suppression with respect to sample also show a match response lower than the non-match, while cells which show match enhancement with respect to sample also show a match response larger than the non-match.

The difference in the response to matching and non-matching stimuli is a result of transient changes in network excitability brought about by the persistent activity. The duration of the transient is mainly controlled by *X*_*E*_, the fraction of slow, NMDA-like recurrent excitatory currents (see also Section *Dependence on persistent activity, response selectivity and current dynamics*). To illustrate this dependence we plot, in Fig. 4B, the average match and non-match responses for three different fractions of slow currents, for a presentation time of 2 seconds. The difference in the responses to match and non-match decays after a time that is proportional to the fraction of slow excitatory currents. Nevertheless, the two responses are still distinguishable after 1 second even for *X*_*E*_ = 0.3. This time interval is significantly longer than the typical presentation times used in experiments, which rarely exceed 500ms.

It has been shown that, for a significant fraction of neurons, tuning curves differ in match and non-match conditions (e.g. [24]). Consistently, we find that the response of most neurons when the stimulus is a match differs from their response when the same stimulus is a non-match. The vast majority of neurons in our simulation exhibits *mixed* effects, that is they show match larger than non-match response for some stimuli, and the opposite behavior for other stimuli (Fig. 4D). In the experiments a significant fraction of cells shows non-mixed effects, i.e.match larger than non-match response or viceversa [8, 11, 24]. The dominance of mixed cells in our simulation is due to the fact that (i) all neurons in the sample we monitor have sharply-tuned persistent activity (i.e. they exhibit persistent activity for a single stimulus); and (ii) the set of stimuli we use to probe them is *optimal*, in the sense that for each neuron there is a stimulus that elicits persistent activity in that neuron. These conditions are likely not to be fulfilled in a real experiment. For instance, recordings from a cell showing persistent activity for all stimuli (which are, typically, not a large number) would result in tuning curves similar to the ones showed in the first two sub-plots in Fig. 3D. Similarly, recordings from a cell showing visual responses but never persistent activity would result in tuning curves similar to the ones showed in the second two sub-plots in Fig. 3D. In general, we would expect the number of mixed cells observed in experiments to increase with the number of stimuli used.

### Protocols with intervening stimuli

We ask next whether the basic mechanism illustrated above is also able to account for the patterns of neural responses observed in DMS tasks in which other stimuli (*distractors*) are presented in between the sample and the test stimulus. In one of such protocols, the so-called *ABBA* protocol [9, 18], the behavioral response is conditional upon the repetition of the sample stimulus (*A*) while the repetition of the distractor (*B*) has to be ignored. In this case, cells were found that showed an enhanced response to the repetition of *A*—the behaviorally relevant match—while they showed suppressed responses (as compared to the match response) for the repeating distractor *B*. At the same time, cells exhibiting match suppression showed larger responses (as compared to the match response) for the repeating distractor [9, 18]. It is worth pointing out that the *parallel mechanisms* hypothesis was formulated in order to account for these experimental observations, in particular for the observation that match enhancement was apparent only upon the repetition of the behaviorally-relevant stimulus [18].

**Figure 5:**
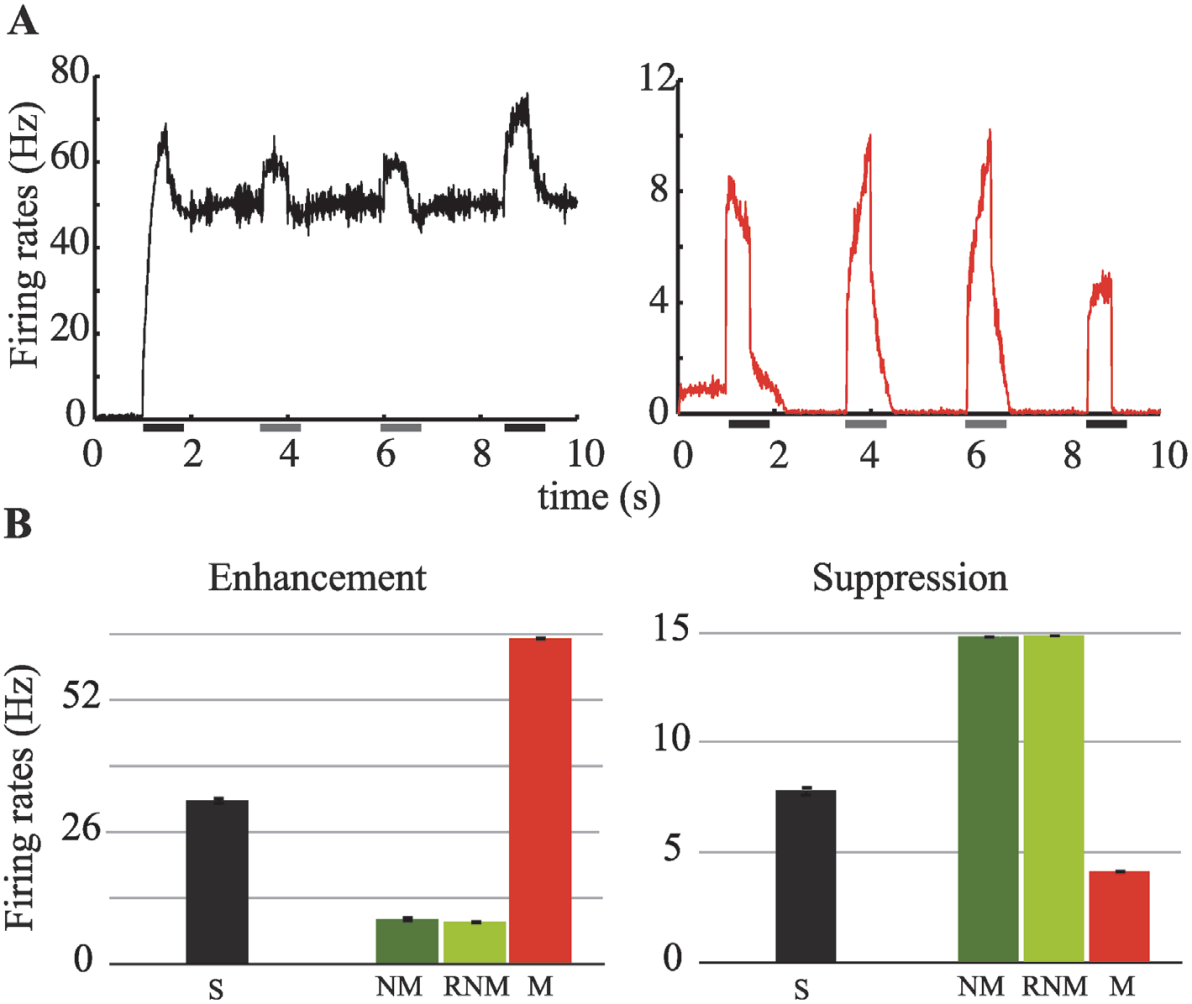
Protocol with repeating distractors. (**A**) Average activity level in the memory representation associated with the sample stimulus (left panel) and in the other representations (right panel). Presentation periods are indicated by the thick horizontal lines: the black lines indicate the sample and the match, while the gray lines indicate the non-match and the repeated non-match i.e. repeating distractors. (**B**) Average response of neural population showing match enhancement (left panel) and match suppression (right panel) for a given stimulus when it is presented as sample, match, non match and repeated non-match (see main text for details). The amplitude of the external inputs are chosen so that the persistent activity elicited by the sample is rarely disrupted by the distractor presentation. We run 20 trials for each stimulus configuration. The error bars indicate the s.e.m.

Our model is able to reproduce the experimental findings described above in a regime where the mnemonic representation activated by the sample stimulus survives the presentation of a distractor with high probability, but is occasionnally disrupted by it. This regime can be obtained by reducing the amplitude of the external inputs during stimulus presentation [32] (see Methods for details). Fig. 5A shows the neural activity in the mnemonic representation associated to the sample stimulus (left panel) and in the remaining representations (right panel). The presentation of the sample activates the corresponding mnemonic representation but also, to a smaller extent, other representations (see Fig. 2). The subsequent presentation of a distractor fails to abolish the mnemonic activity related to the sample (unlike in Fig. 3), although it evokes responses in both the active and the inactive representations.

We then proceed as in the experiment [9, 18]. For each stimulus presented as a sample, we separate the neurons which show enhancement upon repetition (i.e., the ones in the corresponding mnemonic representation) from the neurons which show suppression (i.e., the ones in the remaining representations). Next, we average the activity in these neuronal populations across all trials (with distractors) where the corresponding stimulus is presented as match, non-match and repeated non-match. The results of this analysis are shown in Fig. 5B. As can be seen, neurons eventually showing match enhancement exhibit suppressed responses, as compared to the match response, to repeating distractor presentations (left panel). Similarly, neurons eventually showing match suppression exhibit enhanced responses, again as compared to the match response, to repeating distractor presentations.

**Figure 6:**
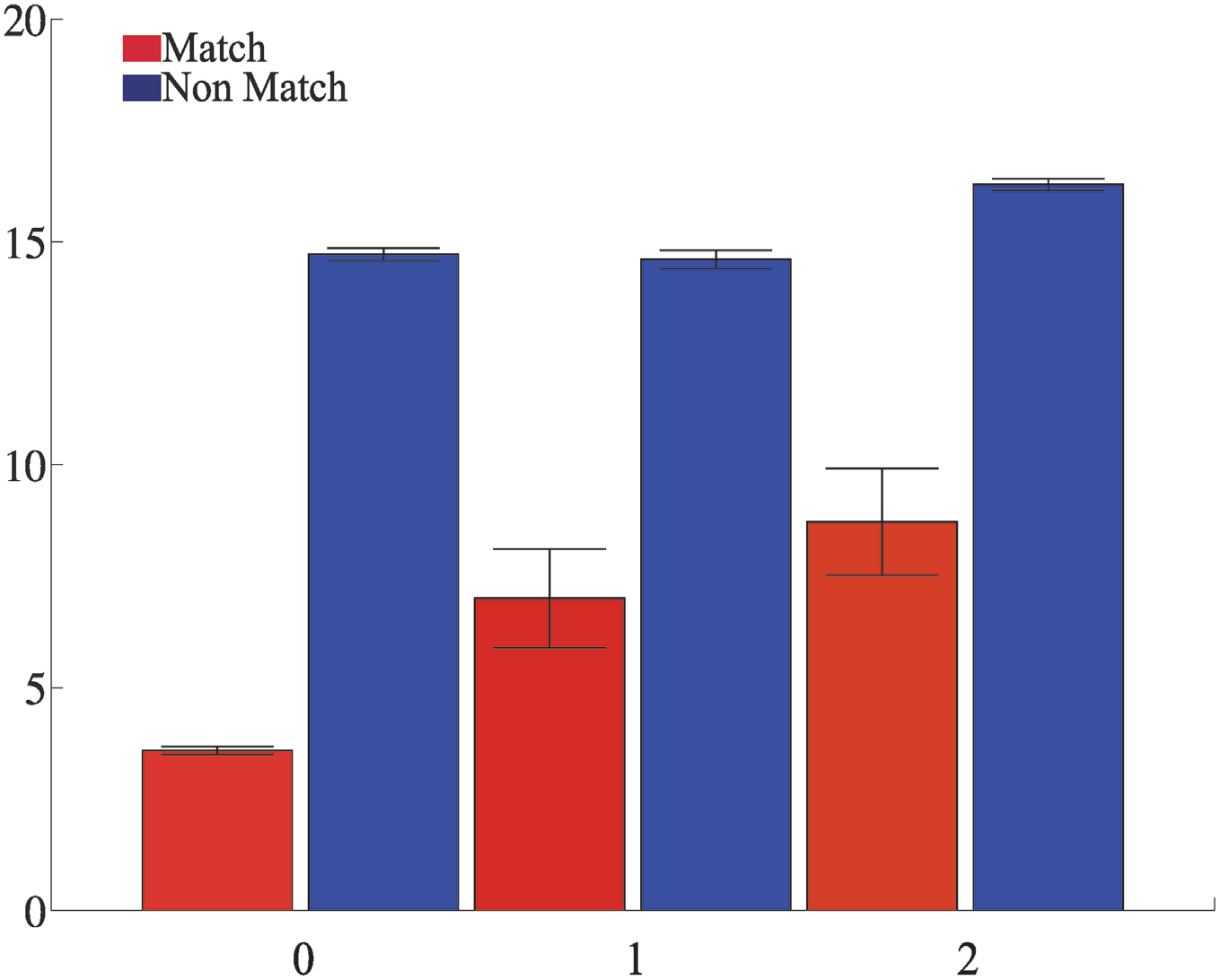
Protocol with non-repeating distractors. Match and non-match responses averaged over the populations of cells exhibiting match suppression as a function of the number of intervening stimuli, i.e. non-repeating distractors. As in Fig. 5, the amplitude of the external inputs are chosen so that the persistent activity elicited by the sample is rarely disrupted by the distractor presentation. We run 20 trials for each protocol. The error bars indicate the s.e.m.

Another common observation in protocols with distractors is that suppression wanes with increasing number of distractors [8]. This finding can also be replicated in the regime where persistent activity survives distractor presentation most of the time, but is occasionally disrupted by it. The amplitude of the population-averaged response depends on whether the mnemonic representation active upon test presentation is the one corresponding to the sample. In particular, if the persistent activity survives the presentation of the distractors, significant suppression will be observed in the majority of cells upon test presentation (see Match-Non Match effects). On the other hand, if the persistent activity is disrupted by the distractor presentation, the fraction of cells exhibiting match suppression will be smaller. The probability that the mnemonic representation associated to the sample is still active upon test presentation is a decreasing function of the number of distractors. In Fig. 6 we plot the average response of cells exhibiting match suppression (i.e. belonging to the mnemonic representations inactive following sample presentation) as a function of the number of intervening distractors. As can be seen, the response in match and non-match trials becomes more similar, i.e. the level of suppression decreases with increasing number of distractors. The same parameter regime can reproduce “standard” match, non-match effects, i.e. when no distractors intervene between sample and match or non-match (“0 distractors” in Fig. 6). Hence, importantly, in our model a unique set of parameters can account for standard match non match protocols, as well as for protocols with intervening stimuli.

### Dependence on persistent activity, response selectivity and current dynamics

We analyze systematically how match effects depend on persistent activity (*PA*), selectivity of the visual responses, and currents dynamics via both a simplified rate dynamics (SRD -that converges to the stationary states described by mean field equations; see Methods) and the spiking network dynamics (SND).

**Figure 7:**
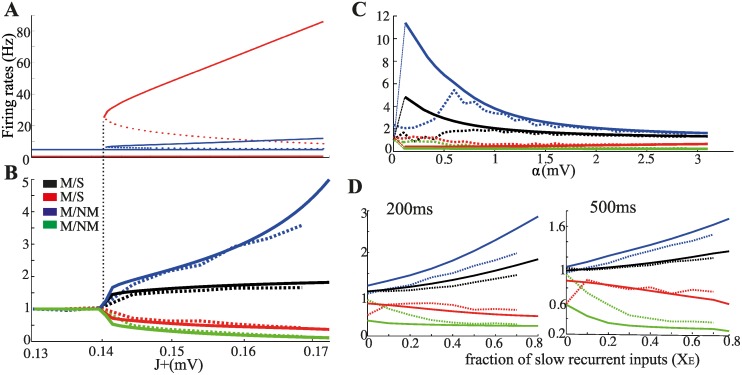
Dependence of match effects on level of persistent activity, selectivity of the responses and current dynamics. (**A**) Persistent activity (red) and inhibitory activity (blue) as a function of *J*_+_. Note for *J*_+_ below a given value the network is unable to sustain persistent activity. The dotted branch of both curves represents unstable solutions. (**B**) Match effects indices as a function of *J*_+_ calculated with SRD (full lines) and SND (dashed lines). Match effects quantitatively increase with increasing *J*_+_, that is with increasing levels of persistent activity. (**C**) Match effect indices *vs* *α*. Match effects are quantitatively more important at low/moderate selectivity levels. (**D**) Match effects indices with SRD (full lines) and SND (dashed lines) *vs* the fraction of slow recurrent inputs (modulated by NMDA-Rs). On the left, indices are calculated by averaging the response over the first 200ms of stimulus presentation; on the right the response is calculated over 500ms, i.e. the entire stimulus duration. See main text for details.

First, we study the dependence of match effects on the level of *PA* by manipulating the synaptic strength between neurons in the same memory representations (*J*_+_), keeping all the other parameters fixed. To quantify match effects we use the enhancement (suppression) index, defined as the ratio between match (*M*) and sample (*S*) response in the active (inactive) memory representation and the match-non match index, defined as the ratio between *M* and non-match (*NM*) response in a memory representation. We find that match effects increase with increasing levels of *PA* (Figs. 7A–B). Neurons in the active memory representation show larger responses to *M* than to *S*, as well as larger responses to *M* than to *NM* (black and blue line in Fig. 7B). The amplitude of both suppression and enhancement increases with increasing *J*_+_ due to the concomitant increase of the level of *PA* (increasing responsiveness in the active representation) and the level of global inhibition (decreasing responsiveness in the inactive representations). In the absence of *PA*, all indices are equal to one, i.e. there is no modulation of response. The simplified rate dynamics (SRD-full lines) give results that are very close to the spiking neurons dynamics (SND-dashed lines).

Next, we study the dependence of match effects on the selectivity of the visual responses by manipulating the difference (*α*) between the average additional input to the memory representation associated to the stimulus presented (*α* + *β*) and the average additional input to the other representations (*β*); all other parameters are kept fixed. We find that match effects are quantitatively more important for low/moderate levels of selectivity, but are nevertheless preserved for strongly selective responses. For *α* close to zero, the response to stimulus presentation is not selective enough to allow the network to activate the corresponding memory representation. In this regime, all indices calculated via the SRD are equal to one (full lines in Fig. 7C). For moderate *α* (0.1 mV < *α* < 0.8 mV), the network is able to activate the memory representation associated with the presented stimulus and, moreover, PA survives the presentation of a *NM*. As a result enhancement effects are quite strong (compare the blue/black with the red/green curves). In the SND, instead, the stimulus presentation could stochastically activate any of the memory representations, thus yielding high fluctuations on the indices (dashed lines in Fig. 7C) and low agreement with SRD. For larger *α*, *PA* is disrupted by the presentation of a *NM*, and enhancement and suppression effects become more comparable. Note that the range of *α* for which *PA* survives the presentation of the *NM* increases with increasing *J*_+_.

In our model, match effects result from modifications of the transient network response following stimulus presentation. Accordingly, decreasing the fraction of recurrent inputs with slow dynamics, and thus speeding up transients, reduces both suppression and enhancement effects (Fig. 7D). Nevertheless, even for relatively small fractions of slow recurrent inputs, match effects are still significant when the responses are averaged over the entire duration of stimulus presentation (i.e., 500ms—right panel in Fig. 7D). This is consistent with experiments showing that match effects are mostly evident during the early response phase [33].

### Effects of heterogeneity in neuronal responses

We have focused so far on a regime in which the sensory and the mnemonic representations of a stimulus are highly correlated. The best stimulus for a neuron is typically the one for which it exhibits persistent activity during the delay period and, therefore, it is also the one which elicits the highest response upon match presentation. Thus, strong responses during the sample presentation will typically be enhanced in the match condition, while poor responses will be suppressed. In this regime, active mnemonic representations lead to a significant *sharpening* of the associated sensory representations upon stimulus repetition, as we have shown. This could be the relevant scenario for sensory-related areas. In higher order areas (e.g., the pre-frontal cortex), however, sensory and mnemonic representations appear to be less correlated. As frequently observed in the data, neurons which show strong selectivity during stimulus presentation do not necessarily show strong selectivity during the delay period. Vice-versa, some neurons have been shown to exhibit weakly selective response, or no response at all during stimulus presentation while developing strong selectivity during the delay period (see, e.g., [9] for a comparison between stimulus-evoked and delay activity in the inferotemporal and prefrontal cortices).

**Figure 8:**
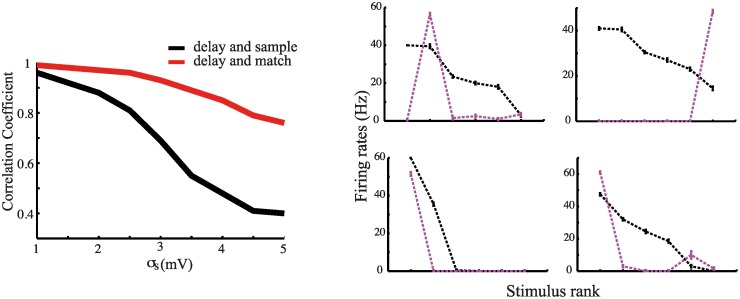
Response heterogeneity. Left panel: Correlation coefficient between the activity averaged across all memory representations during sample and delay (black curve) and during match and delay (red curve) as a function of the variance of the input currents. Right panel: examples of four cells tuning curves at sample -in black- and delay -in magenta- for *σ*_*s*_ = 4 mV. Stimuli are ranked from best to worst according to the responses to the sample.

To investigate the effects of reduced correlation between sensory and mnemonic representations, we manipulated neural response heterogeneity upon stimulus presentation by increasing the variance of the external inputs (*σ*_*s*_—see Methods for details). As *σ*_*s*_ increases, the distribution of inputs to the neurons in the mnemonic representation corresponding to the stimulus being presented becomes more and more similar to the distribution of inputs to the other representations and consequently, the neural responses in these two sets also become similar. As a result, selectivity of neural responses during sample presentation decreases. To quantify the effects of increased variance in the external inputs, we computed for each neuron in the mnemonic representations the correlation (Pearson correlation coefficient) between stimulus-evoked and delay activity across the set of stimuli. The correlation averaged across all neurons in the mnemonic representations is reported in left panel Fig. 8 (black curve). Increasing the variance of the external inputs, steadily and significantly, reduces the correlation between the pattern of activity elicited by the sample and the subsequent pattern of persistent delay activity. This is due to the reduction of the proportion of neurons exhibiting both strong stimulus response and enhanced delay activity. For purpose of illustration, we also show in the right panel of Fig. 8 the tuning curves during sample presentation and delay period for four neurons. As can be seen, both neurons which share the same stimulus preference during sample and delay, and neurons which do not can be found.

Note that increasing the variance of the external inputs has a significantly weaker effect on the correlation between the pattern of activity during the delay period and the pattern of activity elicited upon match presentation (left panel of Fig. 8—red curve). Neurons exhibiting high(low) levels of delay activity will also tend to exhibit strong(poor) responses to match presentation, regardless of the extent of their response at sample. Thus, match effects are largely preserved in the presence of strong heterogeneity in neuronal responses.

## Discussion

In the context of short-term memory tasks which involve the comparison of two subsequent stimuli, e.g. a DMS task, persistent delay activity has often been interpreted as the neural correlate of memory maintenance of the first stimulus. However, maintenance by itself leaves unanswered the question of how the information which is actively being held is retrieved and compared with the stimulus that comes next. We have shown that changes in the spiking rate, as a result of encoding and storage, are naturally accompanied by changes in the neuron’s responsiveness to inputs. The basic mechanism—neurons in high firing states are more responsive than neurons in low firing states—is entirely consistent with both *in vitro* and *in vivo* experiments that compared the response of neurons in UP and DOWN states [25, 34]. In turn, the memory-dependent modifications of the network tuning properties entailed by those changes in single-cell responsiveness parsimoniously account for the experimental phenomenology of match effects exposed by single-cell recordings, as we have shown by theoretical analysis and numerical simulations. Accordingly, our results suggest that persistent activity, along with memory maintenance, might subserve the complementary purpose of endowing responses to stimuli with a history dependence, by filtering out the information which is not consistent with the current state of the network.

### Comparison with experimental data and predictions

By mechanistically linking persistent activity and match effects, our model explains naturally why the latter are observed in the same cortical regions where the former is present (e.g. in ITC: [8, 33, 35–38]; in PPC: [39–41]; in PFC: [9, 11, 17, 24]). Upon stimulus repetition, we find that suppression effects are dominant, involving 80% of cell/stimulus combinations. This is due to two factors: (i) cells have broad selectivity properties during visual response, i.e. they respond to most of the presented stimuli; (ii) delay period activity is sparse, i.e. it involves only a small fraction of the excitatory cells of the network. Both features are consistent with neurophysiological recordings in areas of the temporal and frontal lobe. It has been reported that some cells show complete adaptation, i.e. essentially no response to stimulus repetition, despite vigorous response to novel stimuli [42]. Accordingly, we find cells which, although active at sample, stop firing during match presentation (Fig. 3B). In these cells, as in most of the selective cells in our network, the presentation of a new stimulus elicits a response (Fig. 2B), consistent with the observation that cells that show a modulation of response following stimulus repetition are usually broadly tuned [8, 42].

The model predicts that (i) for a given stimulus, (early) single-cell responses should be positively correlated with the level of delay period activity preceding the test presentation; (ii) the proportion of cells showing match enhancement increases with the proportion of cells showing persistent activity, and the higher the level of persistent activity, the larger the amplitude of the enhancement effect. This is consistent with comparisons between IT and PF cortex: enhancement effects are stronger, in proportion of cells involved and in amplitude, in PFC than in ITC, consistent with the fact that the proportion of cells showing persistent activity during the delay period is larger in PFC [9, 11, 12]. Similarly, both persistent activity and match effects increased significantly after training with a DMS task, although both were found also in naive animals [24]; (iii) repetition produces (transiently) a sparser representation of the stimuli. This is due to the fact that the pattern of persistent activity modifies the transient response to repeated stimuli by enhancing the response of the neurons most active during the delay period while suppressing the response of the other—less active—neurons. In a regime where sensory and mnemonic representations are highly correlated, our scenario therefore provides a mechanistic account for the *sharpening model* [1, 5, 6, 42–45], according to which some but not all neurons that initially respond to a stimulus, show suppression to the repetition of that stimulus, and, most importantly, suppression is stronger for the non preferred stimuli, i.e. neurons showing little or no suppression to a repeated stimulus are highly selective for it. Note that such a mechanism, in which persistent activity acts as a ‘matching filter’ that transiently sharpens the response to the test if it matches the sample, could also potentially account for priming phenomena [46]. When increasing the heterogeneity of the evoked responses, and thus decreasing the correlation between sensory and mnemonic representations, the model also accounts for cells showing suppression instead of enhancement upon repetition of their preferred stimulus (see, e.g., [37]), whose tuning curves would look like those shown in Fig. 8.

Miller *et al.* [8, 9, 18] used protocols with distractors, and found that (i) enhancement is observed for behaviorally relevant matches, but not for repeating distractors; (ii) both suppression and enhancement effects are still present after a few distractors are presented, but decay progressively with the number of distractors. Our model can reproduce these data in a stochastic scenario in which in most cases the distractors do not perturb the active mnemonic representation, but with some non-zero probability erase the memory from the system. This scenario is fully consistent with both the behavioral [8] and the electrophysiological data in PFC indicating resistance to distractors [9]. Electrophysiological evidence for resistance to distractors in ITC is more mixed (e.g., [9]), though we note some degree of resistance to distractors has been found both in ITC (see, e.g., [37, 47]) and in the entorhinal and perirhinal cortices [10, 48].

Going one step forward, the model predicts that any manipulation (e.g., pharmacological) affecting persistent activity should also have significant impact on match effects. Weakening or destroying persistent activity is expected to severely diminish the amplitude of match effects or abolishing them altogether. For example, it has been shown that the enhancement of the GABA-ergic neurotransmitter system, e.g. via benzodiazepines, slows down working memory processes [49, 50] and impedes repetition suppression [51]. Alternatively, boosting persistent activity by reducing the inhibitory feedback [52] is expected to significantly reduce suppression effects, while increasing enhancement effects both in the proportion of cells exhibiting them and in their amplitude at the single-cell level.

### Comparison with other models

The simplest model for match suppression is some form of adaptation (either firing-rate adaptation, or synaptic short-term depression) on sufficiently long time scales [53]. Match enhancement could be accounted for by a separate population of pyramidal cells having predominantly facilitating synapses, as found in PFC [54]. These purely passive mechanisms are however hard to reconcile with evidence that inter-trial intervals erase match effects, as reported by [8]. Our model would be consistent with the fact that inter-trial intervals erase these effects, provided persistent activity is switched off during inter-trial intervals (but see [55]). Note that introducing adaptation and/or short-term synaptic plasticity in our framework would not alter qualitatively the conclusions. Adaptation and short-term depression would tend to decrease quantitatively both enhancement and suppression effects, while short-term facilitation would tend to increase these effects.

A combination of passive and active mechanisms—inspired by the *parallel mechanisms* theory—has been implemented in a recent study by [19], through a network composed of two separate neural populations (one exhibiting enhancement, the other suppression), receiving top-down inputs from a working memory area. The model was shown to be able to reproduce part of the experimental phenomenology reported in [8, 9, 18], but leaves aside a rather critical issue: by assuming two separate sets of cells showing either match enhancement or suppression, the model cannot account for the experimental observation of mixed cells, which show both enhancement and suppression depending on the stimulus presented [24]. We note that, albeit the underlying mechanism is different, the patterns of neuronal activity produced by our model are the same as the ones produced by the Engel and Wang model [19]. Their learning circuit would hence be effective also in our network, providing a biologically realistic read-out mechanism. Another class of models for match effects relies on synaptic modifications induced by stimulus presentations [56, 57]. These mechanisms are not mutually exclusive, and might cooperate to generate strong match signals.

As our main goal in this work was to show that changes in excitability brought about by persistent activity were a viable mechanism for match effects, we have chosen the simplest possible spiking neuron model able to maintain patterns of persistent activity thanks to increased synaptic strength between populations of neurons [26]. In such a model, firing rates in persistent activity are too high and homogeneous, spiking irregularity too low, and the proportion of neurons showing match effects somewhat larger as compared with the experimental data, unless additional features such as short-term plasticity are added (e.g., [58, 59]). We do not expect these additional features to change qualitatively the picture described in this paper, rather we expect them to bring model behavior quantitatively closer to experimental data (see Effects of heterogeneity in neuronal responses).

We note that the general scenario presented above would still hold if attractors are stabilized by other mechanisms (e.g., [60–62]) provided enhanced activity entails increased single-neuron excitability.

### Conclusions

The theory we have presented demonstrates a new functional role for persistent activity beyond temporary memory storage. Our results show how persistent activity can be instrumental in the retrieval of the stored information and, potentially, in the context-dependent encoding of incoming information (see, e.g., [13]). In our implementation, persistent activity is the result of the network possessing multiple steady states of activity (attractors) each manifested by elevated firing rates in stimulus-selective sub-populations of neurons [26, 27]. The fact that the network is in such an attractor modifies the transient response to incoming stimuli; such response could then be exploited by a readout network, which could easily solve any task involving comparisons of sample and match/non-match stimuli. This general scenario would still hold if attractors are stabilized by other mechanisms [60, 61]. Our results also lay the groundwork for uncovering physiological/mechanistic substrates common to different types of mnemonic processing, by suggesting specific neuronal signatures of memory retrieval and possible underlying mechanisms.

## Methods

### Spiking neurons network

In the following we describe the full spiking neurons network simulation used for the results reported in the main text. The behaviour of a single excitatory or inhibitory neuron in the network can be described (below firing threshold) by the dynamics of its membrane potential, which obeys

$$\tau_{E,I}\dot{V_{i}\left(t\right)}=-V_{i}\left(t\right)+I_{i}\left(t\right)$$ (1)

with *i* = 1 … *N*, where *N* = *N*_*E*_ + *N*_*I*_ is the total number of neurons in the network, *τ*_*E*,*I*_ is the integration time constant of the membrane potential for excitatory and inhibitory neurons, and *I*_*i*_ (in mV) is the total current impinging on the (post-synaptic) neuron. When the membrane potential reaches the firing threshold *θ*, upon integration of the incoming current, then a spike is emitted, the membrane potential is reset to a value *V*_*R*_ and the neuron remains refractory for a time *τ*_*arp*_.

The total current arriving to a postsynaptic neuron is due to the activity of its local (pre-synaptic) afferents and to the current elicited by external afferents, e.g. neurons in neighboring cortical areas, namely

$$I_{i}\left(t\right)=I_{i}^{ext}\left(t\right)+I_{i}^{rec}\left(t\right)$$ (2)

where $I_{i}^{ext}(t)$ is the external input current and $I_{i}^{rec}(t)$ is the recurrent current. The recurrent contribution to the post-synaptic current comes from local (pre-synaptic) afferents and is mediated by NMDA, AMPA and GABA receptors, so that

$$I_{i}^{rec}\left(t\right)=I_{i}^{N}\left(t\right)+I_{i}^{A}\left(t\right)-I_{i}^{G}\left(t\right)$$ (3)

where each of the currents follows its own temporal dynamics:

$$\tau_{N}{\dot{I}}_{i}^{N}(t)\;=\;-I_{i}^{N}+X_{E,I}\tau_{E,I}\sum_{j}J_{ij}\sum_{k}\delta (t-t_{k})$$ (4)

$$\tau_{A}{\dot{I}}_{i}^{A}(t)\;=\;-I_{i}^{A}+(1-X_{E,I})\tau_{E,I}\sum_{j}J_{ij}\sum_{k}\delta (t-t_{k})$$ (5)

$$\tau_{G}{\dot{I}}_{i}^{G}(t)\;=\;-I_{i}^{G}+\tau_{E,I}\sum_{j}J_{ij}\sum_{k}\delta (t-t_{k})$$ (6)

where *X*_*E*,*I*_ is the fraction of the charge coming from excitatory afferents which is mediated by NMDA receptors and elicits a slower current dynamics on the post synaptic neuron. The remaining fraction of the charge (1 − *X*_*E*,*I*_) is mediated by AMPA receptors and elicits a faster current. The current coming from inhibitory afferents is mediated by GABA receptors. Upon arrival of a pre-synaptic spike at time *t*_*k*_, the postsynaptic current instantaneously receives a “kick” proportional to the synaptic efficiency *J* (in mV), followed by an exponential decay with time constant *τ*_*syn*_, where *τ*_*syn*_ = *τ*_*A*,*N*,*G*_.

In a regime of *spontaneous activity*, i.e. when no external stimulus is presented to the network,

$$I_{i}^{ext}\left(t\right)=\mu_{i}^{ext}+\sigma_{ext}\sqrt{\tau }\eta_{i}\left(t\right)$$ (7)

where $\mu_{i}^{ext}$ is the value of the external current extracted for each neuron from a Gaussian distribution with mean ${\bar{\mu }}_{E}^{ext}$ and ${\bar{\mu }}_{I}^{ext}$, respectively for excitatory and inhibitory neurons, and variance $\sigma_{BG}^{2}$ (*quenched noise*) while *η*_*i*_(*t*) is a white noise process with < *η*_*i*_(*t*) > = 0 and < *η*_*i*_(*t*)*η*_*j*_(*t*′) > = *δ*_*ij*_ *δ*(*t* − *t*′) uncorrelated from neuron to neuron and *σ*_*ext*_ is the amplitude of the temporal fluctuations around $\mu_{i}^{ext}$ (*fast noise*).

Upon presentation of a stimulus each neuron belonging to the selective populations (i.e. the *memory representations*) receives an external current given by:

$$I_{i}^{ext}\left(t\right)=\mu_{i}^{stim}+\sigma_{ext}\sqrt{\tau }\eta_{i}\left(t\right)$$ (8)

where

$$\mu_{i}^{stim}=\mu_{i}^{ext}+\mu_{i}^{sel}$$ (9)

if *i* belongs to the *selective foreground*, the current elicited by the stimulus on each neuron, $\mu_{i}^{sel}$, is drawn from a Gaussian distribution with mean *α* + *β* and variance $\sigma_{S}^{2}$; if *i* belongs to the *selective background* instead:

$$\mu_{i}^{stim}=\mu_{i}^{ext}+\mu_{i}^{sel}$$ (10)

where $\mu_{i}^{sel}$ is drawn from a Gaussian distribution with mean *β* and variance $\sigma_{S}^{2}$. Those distributions are quenched, so that the presentation of a given stimulus elicits always the same current in a given selective neuron.

The Equations for the membrane potential (Equation 1, together with the condition for spike emission and refractoriness) and the Equations 4–6 and 7 for the currents are integrated using the Euler method with a time step *dt* = 0.1*ms*. The mean value of the external current ${\bar{\mu }}_{E,I}^{ext}$ is calculated using mean-field Equations (see below) such that the background activity is at a chosen value ${\bar{\nu }}_{E}^{sp}$, ${\bar{\nu }}_{I}^{sp}$. The value of the synaptic potentiation *J*_+_ between neurons belonging to the same selective population was chosen to ensure stable persistent activity.

The network has been simulated using the standard protocols of match and non-match trials, as well as protocols in which other stimuli (distractors), repeating or not, are presented in between the sample and the test. All protocols are described below. Figs. 2A. and 3A. show the time course of single match and non-match trials simulated with the spiking neurons network with *α* = 1.5*mV* and *β* = 1.8*mV* (all the other parameters are listed in Table 1). Protocols with intervening stimuli (Figs. 5 and 6) are simulated with the spiking neurons network with *α* = 0.84*mV* and *β* = 1.7*mV* (all other parameters in Table 1). Average responses to stimuli -sample, match, non-match, repeated non-match- are calculated by counting the number of spikes discharged by selective foreground and selective background during the first 200ms of stimulus presentation.

**Table 1:** Network parameters for simplified rate dynamics and spiking neurons simulations. In bold, the parameters used in the mean field analysis.

**Selective populations**	p = 6
**Coding level**	f = 0.05
**Excitatory neurons**	*N*_*E*_ = 1600
**Selective excitatory neurons**	$N_{E}^{sel}=pfN_{E}$ = 480
**Inhibitory neurons**	*N*_*I*_ = 400
**Membrane time const. excit.**	*τ*_*E*_ = 0.02s
**Membrane time const. inhib.**	*τ*_*I*_ = 0.01s
**Threshold membrane potential**	*θ* = 20mV
**External noise excit.and inhib.**	*σ* = 0.75mV
**Standard dev of ${\bar{\mu }}^{ext}$**	*σ*_*BG*_ = 1mV
Standard dev of ${\bar{\mu }}^{stim}$	*σ*_*S*_ = 2mV
**Membrane reset potential excit. and inhib.**	*V*_*R*_ = 10mV
**Refractory period**	*τ*_*arp*_ = 0.0025s
Decay time AMPA currents	*τ*_*AMPA*_ = 0.005s
Decay time NMDA currents	*τ*_*NMDA*_ = 0.05s
Decay time GABA currents	*τ*_*GABA*_ = 0.005s
Fraction of NMDA on excit. neurons	*X*_*E*_ = 0.7
Fraction of NMDA on inhib. neurons	*X*_*I*_ = 0.002
**EPSP on excit.**	*J*_*EE*_ = 0.025mV
**EPSP on inhib.**	*J*_*IE*_ = 2.5*J*_*EE*_
**IPSP on excit.**	*J*_*EI*_ = 3*J*_*EE*_
**IPSP on inhib.**	*J*_*II*_ = 4*J*_*EE*_
**Potentiation**	*J*_+_ = 0.156mV
**Depression**	*J*_−_ = (*J*_*EE*_ − *J*_+_ *f*)/(1 − *f*)
**Excit. spontaneous firing rate**	$\nu_{E}^{sp}\;=\;0.75\text{Hz}$
**Inhib. spontaneous firing rate**	$\nu_{I}^{sp}\;=\;5\text{Hz}$
Time step spiking neurons sim.	*dt* = 0.1ms
Time step simplified rate dynamics sim.	*dt* = 1ms

### Mean-field approach

We solve mean field Equations to find the average firing rates of the network *p*+2 populations in stationary conditions [26]. In particular, we study the network state in the absence of stimulus presentation, i.e. during the spontaneous activity state and during the delay period of a DMS task. The set of parameters used to find the networks stationary states is given in Table 1 (in bold); we chose them to be compatible with realistic cortical anatomy.

#### Spontaneous activity state

At steady state, the average total current ${\bar{\mu }}^{sp}$ impinging on a neuron in the absence of stimulus presentation (spontaneous activity state) depends on the average firing rates of the excitatory and inhibitory populations, according to the following *p* + 2 mean field Equations, where we specify the current contributions to the *p* selective populations, the non-selective background (i.e. excitatory neurons which do not participate in the task) and the inhibitory population

$$\begin{array}{c} {\bar{\mu }}_{k}^{sp}\;=\;{\bar{\mu }}_{k}^{rec}+{\bar{\mu }}_{E}^{ext}\text{with}\\ {\bar{\mu }}_{k}^{rec}\;=\;N_{E}J_{EE}\tau_{E}\left[fg_{+}{\bar{\nu }}_{k}^{sp}+fg_{-}\sum_{j\neq k}{\bar{\nu }}_{j}^{sp}+(1-pf)g_{-}{\bar{\nu }}_{0}^{sp}\right]-N_{I}J_{EI}\tau_{E}{\bar{\nu }}_{I}^{sp}; \end{array}$$ (11)

$$\begin{array}{c} {\bar{\mu }}_{0}^{sp}\;=\;{\bar{\mu }}_{0}^{rec}+{\bar{\mu }}_{E}^{ext}\text{with}\\ {\bar{\mu }}_{0}^{rec}\;=\;N_{E}J_{EE}\tau_{E}\left[f\sum_{k=1}^{p}{\bar{\nu }}_{k}^{sp}+(1-pf){\bar{\nu }}_{0}^{sp}\right]-N_{I}J_{EI}\tau_{E}{\bar{\nu }}_{I}^{sp}; \end{array}$$ (12)

$$\begin{array}{c} {\bar{\mu }}_{I}^{sp}\;=\;{\bar{\mu }}_{I}^{rec}+{\bar{\mu }}_{I}^{ext}\text{with}\\ {\bar{\mu }}_{I}^{rec}=\;N_{E}J_{IE}\tau_{I}\left[f\sum_{k=1}^{p}{\bar{\nu }}_{k}^{sp}+(1-pf){\bar{\nu }}_{0}^{sp}\right]-N_{I}J_{II}\tau_{I}{\bar{\nu }}_{I}^{sp} \end{array}$$ (13)

where *f* is the coding level; ${\bar{\nu }}_{k}$ is the firing rate averaged across all neurons in population *k* (*k* = 1 … *p*), ${\bar{\nu }}_{0}$ and ${\bar{\nu }}_{1}$ are the average firing rates of neurons belonging to the non-selective background and to the inhibitory population. ${\bar{\mu }}_{E,I}^{ext}$, in the absence of any incoming sensory stimulus, is the average current coming from neighboring cortical regions to, respectively, excitatory and inhibitory populations. *g*_+_ and *g*_−_ label, respectively, the strength of synaptic potentiation and depression.

The following condition holds

$$f\;J_{+}+\left(1-f\right)J_{-}=J_{EE}$$

where *J*_+_ = *g*_+_ *J*_*EE*_ and *J*_−_ = *g*_−_ *J*_*EE*_. This condition ensures that the firing rates of the selective populations in spontaneous activity is unchanged as *J*_+_ is varied.

The average firing rates of the *p* + 2 populations are instantaneous functions of the average total currents, according to

$${\bar{\nu }}_{K}^{sp}=\Phi \left({\bar{\mu }}_{K}^{sp},\sigma \right);$$ (14)

where *K* = {*k* = 1 … *p*, 0, *I*} and

$$\Phi_{E,I}\left(\mu ,\sigma \right)={\left[\tau_{arp}+\tau_{E,I}\int_{b}^{a}du\sqrt{\pi }exp\left(u^{2}\right)\left[1+erf\left(u\right)\right]\right]}^{-1}$$

$$a=\frac{\theta -\mu }{\sigma },b=\frac{V_{R}-\mu }{\sigma }$$

is the f-I curve of the leaky integrate-and-fire neuron in the presence of white noise, where erf is the standard error function (see e.g. [63]), *μ* is the total average current impinging on a neuron, *σ* is its temporal fluctuations, *θ* is the firing threshold, *V*_*R*_ is the reset potential and *τ*_*arp*_ is the refractory period.

We assume that the mean external current to the excitatory and inhibitory populations coming from neighboring cortical regions is normally distributed with mean ${\bar{\mu }}_{E,I}^{ext}$ and variance $\sigma_{BG}^{2}$. Consequently, the average firing rates of the *p* + 2 populations become:

$${\bar{\nu }}_{K}^{sp}\;=\;\frac{1}{\sigma_{BG}\sqrt{2\pi }}\int_{-\infty }^{+\infty }\exp\left[\frac{-{\left(\mu_{E}^{ext}-{\bar{\mu }}_{E}^{ext}\right)}^{2}}{2\sigma_{BG}^{2}}\right]\Phi ({\bar{\mu }}_{K}^{rec}+\mu_{E}^{ext},\sigma )d\mu_{E}^{ext};$$ (15)

$${\bar{\nu }}_{I}^{sp}\;=\;\frac{1}{\sigma_{BG}\sqrt{2\pi }}\int_{-\infty }^{+\infty }\exp\left[\frac{-{\left(\mu_{I}^{ext}-{\bar{\mu }}_{I}^{ext}\right)}^{2}}{2\sigma_{BG}^{2}}\right]\Phi ({\bar{\mu }}_{I}^{rec}+\mu_{I}^{ext},\sigma )d\mu_{I}^{ext};$$ (16)

where *K* = {*k* = 1 … *p*, 0}. One has to solve the set of *p* + 2 coupled Equations given by Equations (15–16), where ${\bar{\mu }}_{K}^{rec}$ and ${\bar{\mu }}_{I}^{rec}$ are given by Equations (11–13), to find the average firing rates and the average total currents for each population. Having set all the parameters according to Table 1, one still needs to set the value of the external currents ${\bar{\mu }}_{E,I}^{ext}$. We choose ${\bar{\mu }}_{E,I}^{ext}$ in order to have fixed average spontaneous rates:

$${\bar{\nu }}_{k}^{sp}={\bar{\nu }}_{0}^{sp}=0.75Hz;$$ (17)

$${\bar{\nu }}_{I}^{sp}=5Hz;$$ (18)

with *k* = 1 … *p*, and solve the system of coupled Equation via the Newton-Raphson method.

#### Delay period and persistent activity

In the spontaneous activity state the network settles in a “symmetric state” in which all the *pfN*_*E*_ cells belonging to the selective populations fire on average at the same rate ${\bar{\nu }}^{sp}$. By contrast, during the delay period following the presentation of a stimulus, a fraction of the *pfN*_*E*_ selective cells keeps firing persistently even when the external stimulus is removed. We refer to this population of cells as the *selective foreground* (or the active memory representation). The remaining selective cells fall back to spontaneous activity and we refer to them as the *selective background* (or the inactive memory representations). The proportion of cells showing persistent activity during the delay period depends on the strength of the synaptic potentiation *J*_+_ and on the level of activity of each selective population at stimulus offset. In our model, the presentation of an external stimulus activates all the *p* selective populations, although to a different extent (see Fig. 1C. in the main text). There are *p* possible scenarios in which *γ* = 1 up to *γ* = *p* populations receive the highest current elicited by stimulus presentation. We set in a scenario in which *γ* = 1, hence only one of the selective populations is activated the most by the external stimulus while the other *p*−1 are all responding equally less. Consequently, when the external stimulus is removed, if *J*_+_ is large enough, only one selective population, i.e. the selective foreground, will fire persistently; all the other, i.e. the selective background, will fall back to spontaneous activity.

In this situation the system can be described by four functionally different populations of cells: the selective foreground whose average firing rate is labelled by ${\bar{\nu }}_{sf}$, the selective background which fires on average at ${\bar{\nu }}_{bf}$, the non-selective background, ${\bar{\nu }}_{0}$, and the inhibitory population ${\bar{\nu }}_{I}$. Therefore, the average currents received by each of them, once the external stimulus is removed, can be written as

$$\begin{array}{c} {\bar{\mu }}_{sf}\;=\;{\bar{\mu }}_{sf}^{rec}+{\bar{\mu }}_{E}^{ext}\text{with}\\ {\bar{\mu }}_{sf}^{rec}\;=\;N_{E}J_{EE}\tau_{E}[f{\bar{\nu }}_{sf}(g_{+}+(\gamma -1)g_{-})+f{\bar{\nu }}_{sb}(p-\gamma )g_{-}+(1-pf)g_{-}{\bar{\nu }}_{0}]-N_{I}J_{EI}\tau_{E}{\bar{\nu }}_{I}; \end{array}$$ (19)

$$\begin{array}{c} {\bar{\mu }}_{sb}\;=\;{\bar{\mu }}_{sb}^{rec}+{\bar{\mu }}_{E}^{ext}\text{with}\\ {\bar{\mu }}_{sb}^{rec}\;=\;N_{E}J_{EE}\tau_{E}[f{\bar{\nu }}_{sf}\gamma g_{-}+f{\bar{\nu }}_{sb}(g_{+}+(p-\gamma -1)g_{-})+(1-pf)g_{-}{\bar{\nu }}_{0}]-N_{I}J_{EI}\tau_{E}{\bar{\nu }}_{I}; \end{array}$$ (20)

$$\begin{array}{c} {\bar{\mu }}_{0}\;=\;{\bar{\mu }}_{0}^{rec}+{\bar{\mu }}_{E}^{ext}\text{with}\\ {\bar{\mu }}_{0}^{rec}\;=\;N_{E}J_{EE}\tau_{E}[f{\bar{\nu }}_{sf}\gamma +f{\bar{\nu }}_{sb}(p-\gamma )+(1-pf){\bar{\nu }}_{0}]-N_{I}J_{EI}\tau_{E}{\bar{\nu }}_{I}; \end{array}$$ (21)

$$\begin{array}{c} {\bar{\mu }}_{I}\;=\;{\bar{\mu }}_{I}^{rec}+{\bar{\mu }}_{I}^{ext}\text{with}\\ {\bar{\mu }}_{I}^{rec}=\;N_{E}J_{IE}\tau_{I}[f{\bar{\nu }}_{sf}\gamma +f{\bar{\nu }}_{sb}(p-\gamma )+(1-pf){\bar{\nu }}_{0}]-N_{I}J_{II}\tau_{I}{\bar{\nu }}_{I} \end{array}$$ (22)

and the average firing rates in each populations are given by Equations (15–16) where *K* = {*sf*, *sb*, 0}.

For each fixed value of *J*_+_ and *γ*, one can find numerically the stable solutions (spontaneous state and persistent activity state) of the system of four coupled Equations by appropriately setting the initial conditions and then solving via the Newton-Raphson method.

### Simplified rate dynamics

Via the mean field approach described above we are able to completely define the attractor landscape of the network, i.e. the available stationary states of its dynamics. As a next step, in order to study the network’s transient response during *sample*, *match* and *non*−*match* presentations, we use a simplified rate dynamics on the average population currents. We solve the dynamics for each epoch of a *match* and *non*—*match* trial, i.e spontaneous activity, sample presentation, delay period, match/non-match presentation (see Protocols for the time duration of each epoch). Such model, without being as realistic and detailed as a network of spiking neurons, allows us to explore the relevant parameters’ space in a less time consuming way. The rate dynamics is not exact, but becomes a good approximation when the firing rates are low [64] and gives in general a good approximation of the spiking network dynamics (see Fig. 5 in the main text).

In accordance with the spiking neuron model (see the above section), we consider the different kinetics of the AMPA, GABA, and NMDA receptors to describe the dynamics of the average current for each of the four functionally relevant populations in the network. Hence, the total average current afferent on each population is given by

$${\bar{\mu }}_{K}=I_{K}^{N}+I_{K}^{A}-I_{K}^{G}+{\bar{\mu }}_{K}^{ext}$$ (23)

where *K* = {*sf*, *sb*, 0, *I*}, ${\bar{\mu }}_{K}^{ext}={\bar{\mu }}_{E}^{ext}$(${\bar{\mu }}_{I}^{ext}$) for the excitatory(inhibitory) populations and *I* ^*N*^(*I* ^*A*^) is the contribution to the average current coming from excitatory afferents and mediated by NMDA(AMPA), while *I* ^*G*^ is the contribution to the average current coming from inhibitory afferents and mediated by GABA receptors. Each of these components evolves in time according to its own time constant

$$\begin{array}{ccc} \tau_{N}{\dot{I}}_{K}^{N} & = & -I_{K}^{N}+X_{K}{\bar{\mu }}_{K}^{rec_{E}};\\ \tau_{A}{\dot{I}}_{K}^{A} & = & -I_{K}^{A}+\left(1-X_{K}\right){\bar{\mu }}_{K}^{rec_{E}};\\ \tau_{G}{\dot{I}}_{K}^{G} & = & -I_{K}^{G}+{\bar{\mu }}_{K}^{rec_{I}} \end{array}$$

where ${\bar{\mu }}_{K}^{rec_{E}}\;+\;{\bar{\mu }}_{K}^{rec_{I}}\;=\;{\bar{\mu }}_{K}^{rec}$ and ${\bar{\mu }}_{K}^{rec}$ are defined in Equations 19–22.

As for the spiking network, *τ*_*N*_, *τ*_*A*_ and *τ*_*G*_ are, respectively, the decay time constants of NMDA, AMPA and GABA currents, while *X*_*sf*,*sb*,0_(*X*_*I*_) is the fraction of NMDA current on the excitatory(inhibitory) population and *γ* = 1. Note that, as before, the total average currents defined in 23 depend on the average firing rates of each population according to

$${\bar{\nu }}_{k}=\frac{1}{\sigma_{BG}\sqrt{2\pi }}\int_{-\infty }^{+\infty }\exp\left[\frac{-{\left(\mu_{k}^{ext}-{\bar{\mu }}_{k}^{ext}\right)}^{2}}{2\sigma_{BG}^{2}}\right]\Phi \left(I_{k}^{N}+I_{k}^{A}-I_{k}^{G}+\mu_{k}^{ext},\sigma \right)d\mu_{k}^{ext};$$

Having introduced slow and fast current dynamics in the simplified rate model allows to have a good agreement with the spiking neurons model also during stimulus presentation. Responses to sample, match and non-match are calculated either by averaging across the responses of all selective populations over the first *T* = 200*ms* of stimulus presentation (unless otherwise stated) or by averaging separately across selective foreground and selective background.

### Protocols

We use *p* = 6 different stimuli, corresponding to the *p* = 6 *memory representations* in the network. All trials begin with a pre-stimulus interval (1s) in which no external stimulus is presented and the network is in a regime of *spontaneous activity*. Subsequently, a first stimulus is presented for 0.5s (*sample*). In *match* trials, after a delay period of 0.7s in which the external stimulus is removed, a stimulus identical to the sample is presented for 0.5s (*match*). In *non*—*match* trials, after the delay period (0.7s), a stimulus different from the sample is presented for 0.5s (*non*—*match*). In protocols with intervening non-repeated stimuli, sample presentation is followed, after a delay period, by either 1 or 2 -different- distractors and then by a test stimulus, which could be either a match or a non-match to the sample. In protocols with intervening repeated stimuli -i.e. the ‘ABBA’ type of trials- the sample is followed, after a delay period, by 2 repeating distractors and by a final match to the sample. Stimulus and delay period durations are kept the same as in “simple” match/non-match trials.

### Sparseness index

As in [30], we used the following index as a measure of selectivity

$$S=\frac{1-A}{1-\frac{1}{n}}$$

where

$$A=\frac{{\left(\sum_{i}^{n}\nu_{i}/n\right)}^{2}}{\sum_{i}^{n}\left(\nu_{i}^{2}/n\right)}$$

and *n* is the number of stimuli, *ν*_*i*_ are the mean firing rates to a set of stimuli. S takes values between 0 and 1, so that

$$\begin{array}{ccc} S=0\;\;\text{when}\;\;\nu_{i} & = & \nu \;\;\text{for}\;\text{all}\;\text{i}\;\;(A=1)\\ S=1\;\;\text{when}\;\;\nu_{i} & = & \nu \;\;\text{and}\;\;\nu_{j\neq i}=0\;\;\left(A=1/n\right) \end{array}$$
